# An unusual presentation of invasive aspergillosis with submandibular swelling in a 49‐year‐old man with end‐stage renal disease: A case report

**DOI:** 10.1002/rcr2.905

**Published:** 2022-01-19

**Authors:** Phool Iqbal, Sinda Dakhlia, Sara Seife Hassen, Salah Mahdi

**Affiliations:** ^1^ Department of Internal Medicine Hamad General Hospital, Hamad Medical Corporation (HMC) Doha Qatar; ^2^ Critical Care Department Hamad Medical Corporation Doha Qatar; ^3^ Department of Internal Medicine Al Khor Hospital, Hamad Medical Corporation (HMC) Al Khor Qatar

**Keywords:** end‐stage renal disease, invasive aspergillosis, submandibular swelling

## Abstract

Invasive aspergillosis (IA) is a fatal disease and is related to immunocompromised patients like HIV, solid organ/allogeneic stem cell transplant patients, patients on immunosuppressive therapy or chemotherapy agents, burn patients and malnourished patients. Diagnosis is challenging due to the non‐specific nature of symptoms. Usually, the patient presents with fever and respiratory symptoms such as cough and haemoptysis. We present a case of IA in a 49‐year‐old man with end‐stage renal disease who presented with fever and developed a submandibular swelling. Further imaging studies supported the possibility of having underlying IA and accordingly he was promptly treated with systemic antifungal therapy with good response. As per our knowledge, IA presenting as submandibular swelling has not been reported in the literature. Our main aim is to highlight the significance of early diagnosis and management in such a rare presentation associated with a life‐threatening condition like IA.

## Graphical abstract

Invasive aspergillosis (IA) is a fatal disease and is related to immunocompromised patients. Diagnosis is challenging due to the non‐specific nature of symptoms. We present a case of IA in a 49‐year‐old man with end‐stage renal disease who presented with fever and developed a submandibular swelling.

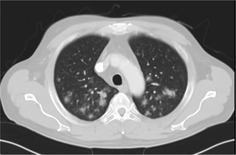

## INTRODUCTION

Invasive aspergillosis (IA) is most commonly seen in immunocompromised patients. Our article describes a case of IA in a patient with hypertension and end‐stage renal disease (ESRD) who initially presented with high‐grade fever with no obvious source on clinical examination. He was investigated for underlying source of infection; however, later during the hospital course, he developed painless submandibular swelling and had severe sepsis. His computed tomography (CT) of the thorax, abdomen and pelvis was remarkable for multiple halo sign shadows in the lung parenchyma correlating with IA. This diagnosis is rare in ESRD; IA has previously been reported in patients on chemotherapy or with haematological malignancy or following solid organ transplant and allogeneic stem cell transplant.^1^

## CASE REPORT

**FIGURE 1:**
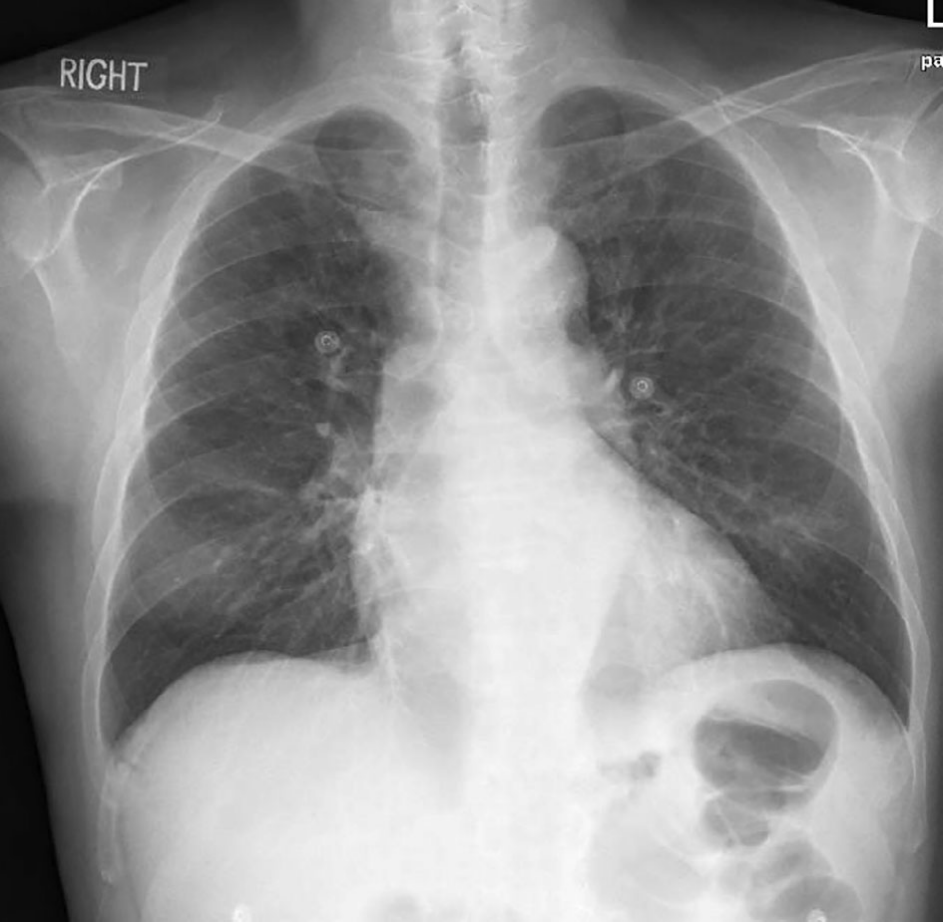
Chest x‐ray unremarkable for any consolidation or any infiltrates

A 49‐year‐old Filipino gentleman, known to have long‐standing hypertension that led to ESRD, on haemodialysis through an arteriovenous fistula presented to the emergency department with high‐grade fever. There was no history of respiratory, gastrointestinal or urinary symptoms as well as any recent travel or sick contact. On physical examination, he was having high‐grade fever (temperature 39°C), normal blood pressure, the skin around the central catheter was mildly tender, but no erythema or swelling was observed, and the rest of systemic examination was unremarkable. A nephrologist was consulted, and it was decided to remove the central line catheter as a possible source of infection. Initial blood investigations revealed anaemia, leukopenia, thrombocytopenia and mild decrease in neutrophils, as shown in Table 1. Sepsis work up for bacterial, mycobacterial and fungal infection, including blood, urine, sputum, to catheter tip cultures, and novel coronavirus disease 2019 (COVID‐19) screening was sent. A chest x‐ray (XR) was normal as shown in Figure 1. He was started on broad‐spectrum antibiotics with a renal adjusted dose of intravenous (IV) vancomycin, considering central line catheter‐associated blood infection after consulting an infectious disease specialist. His blood cultures and septic workup revealed no organisms to explain his febrile presentation. The culture of the central line catheter tip showed no growth of organisms.

**TABLE 1:** Laboratory investigations during the period of hospital stay

	September 2020	October 2020	October 2020	Normal values
Time period	Week 0	Week 2	Week 4	
White blood count	1.7 ↓	0.9↓↓	7.3	4–10 × 10^3^/μl
Haemoglobin	8.7↓	7.5↓↓	9.4	13–17 g/dl
Platelet	150↓	98↓↓	184	15–400 × 10^3^/μl
Neutrophil count	1.2↓	0.5↓↓	6.2	2–7 × 10^3^/μl
Lymphocyte count	0.3↓	0.2↓↓	1.0	1–3 × 10^3^/μl

**FIGURE 2:**
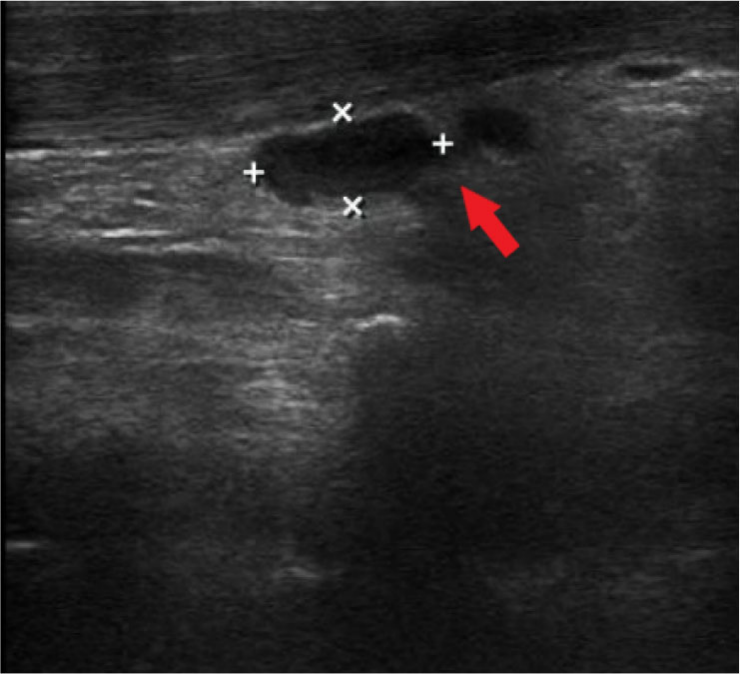
Right submandibular lymph node with mild inflammatory changes

During his hospital stay, he continued to have low‐grade fever despite completing a total of 14 days of IV antibiotic. At day 17 of hospitalization, his temperature spiked to 38.3°C. Clinical examination was unremarkable. Repeat sepsis workup was negative, including screening for HIV, hepatitis B and C, and cluster of differentiation count subset. Repeat chest XR was normal as well. Transthoracic and transesophageal echoes were negative. He was treated with IV piperacillin‐tazobactam and vancomycin but with no adequate response. Blood investigations showed pancytopenia (Table 1). His blood pressure gradually started to decline in the range of 100/60 to 90/60 mmHg, which responded well to fluid resuscitation. On day 21, the patient developed sepsis, mild facial puffiness and a localized submandibular swelling. The swelling was mildly tender, not erythematous, hot or fluctuating. Oral cavity examination was unremarkable as well. There were no other body swellings or generalized lymphadenopathy. Ultrasound of the neck revealed inflammatory changes in the left submandibular lymph node suggestive of ongoing infection as shown in Figure 2.

**FIGURE 3:**
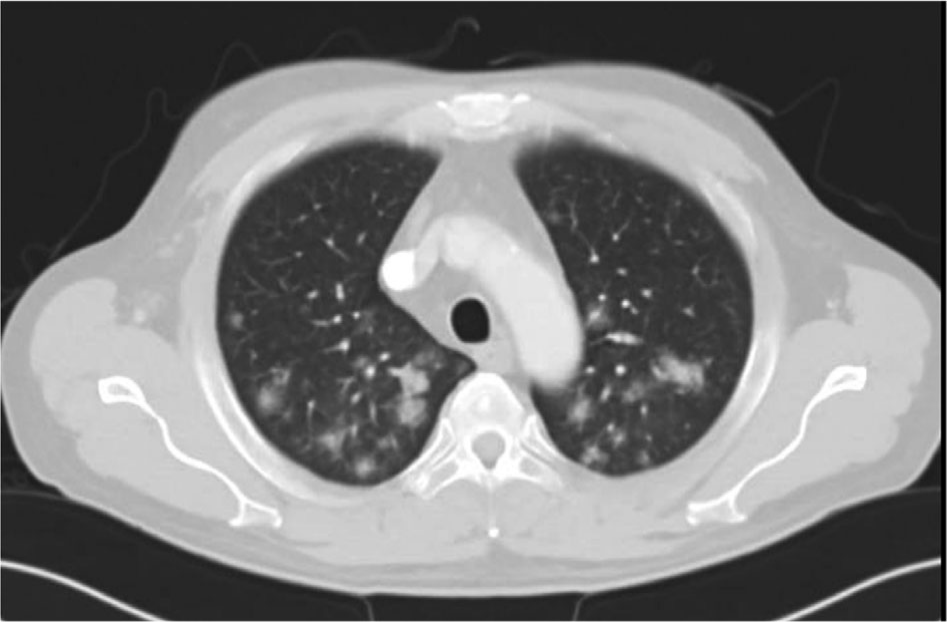
Computed tomography scan of the thorax revealing multiple halo sign shadows in the lung field

In view of the persistent fever and obscure source of infection, CT scan of the neck, chest, abdomen and pelvis (pan CT) along with consultation with the surgical team was decided for possible excisional biopsy of the mass, to look for hidden collection or malignancy. However, the patient developed breathing difficulty with increasing facial puffiness but maintaining reasonable O_2_ saturation, and hence, was admitted to the intensive care unit (ICU) for observation of any airway compromise. Pan CT result was remarkable for multiple halo signs in the lung field suggestive of disseminated fungal infection (Figure 3); the diagnosis was supported by a positive blood test for galactomannan antigen.

IV caspofungin was included as recommended by infectious disease team. The neck swelling subsided after the commencement of antifungal therapy with significant resolution of the submandibular mass within 7 days; his haemoglobin as well as platelets and neutrophil counts improved (Table 1). Therefore, surgeons did not proceed with the excisional biopsy. An infectious disease specialist and nephrologist were on board with the internal medicine team for managing this patient. He received a total of 14 days of IV antifungal therapy. The patient continued to be afebrile during his further hospital stay. He was discharged on oral voriconazole for further 2 weeks and was followed up for 1 month by an infectious disease specialist. He remained stable throughout the follow‐up, with resolution of pancytopenia.

## DISCUSSION

The incidence of invasive fungal infection varies from 2% to 36.5% out of which 40%–50% are caused by Aspergillus species. In the largest reported series, aspergillosis is found in around 0.7% of children with cancer.^2^ The overall case‐fatality rate was systematically reviewed by Lin et al. in 2001 and was found to be 58%.^3^

Conditions that compromise the immune system predispose patients for IA and may result in high mortality rates.^4^, ^5^ IA can occur in a number of immunocompromised patients including bone marrow transplant recipients, with haematological malignancy, T‐cell‐depleted grafts, glucocorticoids and on immune suppressive drugs such as tyrosine kinase inhibitors. It can occur in AIDS patients, patients with primary immunodeficiency or those receiving immunosuppressive medications such as tumour necrosis factor‐a blockers.^6^ As for liver and lung transplant recipients, neutropenic patients and extensively burned patients, they should be housed in high efficiency particulate air‐filtered environments if possible as prophylaxis for aspergillosis.^7^ On the other hand, IA may occur in immunocompetent hosts with no underlying lung disease such as extended ICU stay (>21 days), haemodialysis, liver cirrhosis, chronic obstructive pulmonary disease, burn injury, diabetes, malnutrition and influenza infection.^4^, ^8^ For the septic patient, often in the ICU, there is an explanation for susceptibility to aspergillosis that involves immunosuppression, induced by sepsis itself which was the case in our patient.^8^ Our patient was a case of ESRD due to hypertensive nephropathy, whose initial presentation was fever complicated by sepsis and neutropenia. With the help of multiple sub‐specialties, infectious disease, haematology, internal medicine and critical care, the patient was labelled and treated successfully as a case of IA. There was no evidence of other infections (e.g., viral), autoimmune disease or malignancy from our investigations therefore bone marrow examination was not performed. The patient had resolution of his pancytopenia, aside from normocytic anaemia consistent with ESRD.

As per the European Organization for Research and Treatment of Cancer and the Mycoses Study Group Education and Research Consortium, diagnostic criteria proving invasive pulmonary aspergillosis involve the microscopic analysis of sterile material through histopathological, cytopathological and microscopic examination, and for probable diagnosis, three factors need to be met: host factors (including history of neutropenia which was met by our patient), clinical features (which showed the halo sign on CT) as well as mycological criteria (such as galactomannan test which was positive in our patient). In our patient, bronchoalveolar lavage fluid was negative for tuberculosis or any fungal infection and blood cultures were negative as well.^9^

The diagnosis of fungal infections may be missed or delayed due to non‐specific clinical signs in patients with ESRD, approximately 25%–33% of patients initially have no symptoms attributable to invasive pulmonary aspergillosis.^1^ For this reason, empiric treatment in patients with strongly suspected IA is warranted while a diagnostic evaluation is conducted.^3^ In our patient's case, he was started on voriconazole with antibiotics as soon as sepsis with neutropenia was established prior to obtaining cultures or biopsy of the lump. In IA, therapy with voriconazole is reported to be better tolerated and more efficacious than amphotericin B.^10^ At discharge, he was prescibed oral antifungal therapy and completed the course. His clinical symptoms gradually improved upon follow up. Oral administration of antifungal medications should be considered in stable patients of IA.^11^ As for fungal therapy, an observational study in patients with acute myeloid leukemia‐Myelodysplatic syndrome receiving high‐dose chemotherapy showed that early diagnosis of IA and treatment with oral voriconazole therapy from day 1 can result in a 12‐week overall mortality rate of 22% in adult patients (age > 17 years).^12^

In addition, the duration of therapy has no clear‐cut evidence‐based guidelines. Thus, expert recommendation is for treatment through symptomatic or radiological resolution.^2^ However, because IA has long been known to result in residual defects due to tissue infarcts, it has a propensity to reactivate, especially in the setting of continuous immunosuppression causing a subacute or even chronic infection.^13^

Rare examples of IA are ocular aspergillosis as well as Aspergillus osteomyelitis which is an entity affecting bones including the spine (49%), skull base, paranasal sinuses and jaw (18%), ribs (9%) and long bones (9%).^6^ However, the presentation as submandibular swelling in association with IA has not been reported before in the literature.

We aim to highlight an association of a rare presentation with IA which is a fatal disease if not treated in a timely manner. Keen diagnostic approach for suspected cases and empiric treatment with systemic antifungal therapy may warrant a good prognosis in such challenging clinical scenarios.

## CONFLICT OF INTEREST

None declared.

## AUTHOR CONTRIBUTION

Phool Iqbal: History and physical, manuscript writing, literature review, review and approval of the final manuscript. Sinda Dakhlia: Manuscript writing, literature review, review and approval of the final manuscript. Sara Seife Hassen: History taking and physical examination of the patient, manuscript writing, literature review, obtained informed written consent, review and approval of the final manuscript. Salah Mahdi: Case selection, literature review, manuscript writing, prescribing medicine, clinical follow‐up.

## ETHICS STATEMENT

The authors declare that appropriate written informed consent was obtained for publication of this manuscript and accompanying images.

## Data Availability

Data sharing is not applicable to this article as no new data were created or analysed in this study.
